# Review of the Genus *Mictis* (Hemiptera: Heteroptera: Coreidae: Coreinae) from China, with Description of a New Species †

**DOI:** 10.3390/insects16111099

**Published:** 2025-10-28

**Authors:** Harvey Xun, Kun Jiang, Weibing Zhu

**Affiliations:** 1 Independent Researcher, No. 8 Courtyard, Shunsha Road, Beijing 102200, China; xunharvey@163.com; 2 Collaborative Innovation Center of Recovery and Reconstruction of Degraded Ecosystems in Wanjiang Basin Co-Founded by Anhui Province and Ministry of Education, School of Ecology and Environment, Anhui Normal University, Wuhu 241000, China; jiangkunnk@163.com; 3 CAS Center for Excellence in Molecular Plant Sciences, Chinese Academy of Sciences, Shanghai 200032, China

**Keywords:** Hemiptera, Coreoidea, taxonomy, *Mictis*, oriental region

## Abstract

**Simple Summary:**

The classification of *Mictis*, a group of large and widely distributed insects, has long been unclear, with unresolved issues in taxonomy. This study clarifies the taxonomy of Chinese *Mictis* species, proposing two new synonyms (*Mictis serina* = *Mictis fuscipes* **syn. n.**, *Mictis longicornis* = *Mictis tuberosa* **syn. n.**) and one new combination (*Ochrochira falloui* **comb. n.**). Additionally, a new species, *Mictis arcuata* **sp. n.**, is described. To aid identification, an illustrated key and DNA barcode data are provided.

**Abstract:**

Species of the genus *Mictis* Leach, 1814, notable for their large body size and wide distribution, have attracted significant attention in physiological and phylogenetic studies. However, taxonomic issues surrounding these insects have long been overlooked, with the validity and taxonomic status of several species remaining unresolved. This study systematically reviews the nomenclatural and taxonomic issues of the genus *Mictis* within China, resulting in the proposal of two new synonyms and one new combination: *Mictis serina* (Dallas, 1852) = *Mictis fuscipes* (Hsiao, 1963) **syn. n.**, *Mictis longicornis* (Westwood, 1842) = *Mictis tuberosa* (Hsiao, 1965) **syn. n.**, and *Ochrochira falloui* (Reuter, 1888) **comb. n.** All known *Mictis* species from the region are diagnosed, and a new species, *Mictis arcuata* **sp. n.**, is described. An identification key and DNA barcoding data for the *Mictis* species are provided. The intra-specific chromatic variation and distribution of the genus are also discussed.

## 1. Introduction

Mictini Amyot & Serville, 1843 (Hemiptera: Heteroptera: Coreidae) is a group of herbivorous insects characterized by their large size and incrassate, spiny hind femora [1,2]. Highly diverse, Mictini is distributed across Asia, Africa, and Oceania, with habitats closely linked to their host plants [2,3,4]. Currently, Mictini includes 48 genera and approximately 380 species, making it one of the most species-rich tribes within the Coreoidea subfamily [5].

Among the Mictini, the genus *Mictis* Leach, 1814 is particularly notable due to its morphological diversity and wide distribution. Established as the type genus of Mictini, with *Mictis profana* Fabricius, 1803 as the type species, *Mictis* includes 21 Asian species, 3 Oceanian species, and 1 African species [5,6]. *Mictis* is a relatively large genus, displaying greater variation than many other Mictini genera. The genus is easily distinguished from closely related genera by a large median tubercle at the common boundary of abdominal segments III and IV, and a small spine on each side of abdominal segment III [2]. Despite its physiological and systematic significance, the taxonomy of *Mictis* remains poorly resolved, necessitating a revision of its nomenclature and classification [7,8,9].

This study reviews the taxonomy of *Mictis* in China, providing a comprehensive overview of the genus. We propose one new combination and two new synonymies, describe a new species, and provide an identification key for the Chinese species of *Mictis*. Additionally, we summarize and discuss the systematic relationships and distribution of *Mictis*.

## 2. Materials and Methods

### 2.1. Material Depository

Specimens examined in the present study are deposited in the following collections:

BMNH—Natural History Museum, London, United Kingdom.
CHX—personal collection of Harvey Xun, Beijing, China.
CMO—personal collection of Mengteng Ouyang, Nanchang, China.
CZZ—personal collection of Zhichao Zhang, Dalian, China.
EANU—School of Ecology and Environment, Anhui Normal University, Wuhu, Anhui, China.
IZCAS—National Animal Collection Resource Center, Institute of Zoology, Chinese Academy of Sciences, Beijing, China.
MNHN—Muséum national d’Histoire naturelle, Paris, France.
NKUM—Nankai University, Tianjin, China.
SHEM—Shanghai Entomological Museum, Shanghai, China.
UMO—University Museum of Oxford, Oxford, United Kingdom.
ZMUC—Zoological Museum, University of Copenhagen, Copenhagen, Denmark.

### 2.2. Morphological Study

All newly collected specimens were preserved in 98% ethanol (EtOH), and some males were dried and pinned after the removal of the genital capsule. Morphological terminology follows [6,10]. The male genital capsule was treated with 10% sodium hydroxide in a 75 °C water bath for two hours to dissolve muscle tissue. Photographs of the habitus were taken using a Nikon z50 camera with a 65 mm F2.8 Macro lens (Nikon, Tokyo, Japan). Photographs of male genitalia were taken using an Olympus EM5 Mark III camera equipped with an Olympus M.Zuiko Digital ED 60 mm f/2.8 Macro lens (Olympus, Hachioji, Tokyo, Japan) and a Nikon D610 camera with a tube lens and a Mitutoyo M Plan Apo 10× objective lens (Mitutoyo, Kawasaki, Japan). The depth of field overlay was achieved using the WeMacro automatic focus stacking rail, and the image stacking was completed with Helicon Focus 8.0.4 Pro. Photos were enhanced using Adobe Photoshop 2024 and Adobe Lightroom Classic. The distribution map was made with QGIS 3.42.2.

Measurement criteria in millimeters (mm) are as follows: body length (BL): the length between the apex of the mandibular plates to the hemelytral apex along the midline; head length (HL): the length between the apex of the mandibular plates and the basal margin of the vertex along the midline; head width (HW): the widest part of the head (including eyes); interocellar space (IS): the distance between ocelli; antennal segments I (AS I): the length of antennal segments I; antennal segments II (AS II): the length of antennal segments II; antennal segments III (AS III): the length of antennal segments III; antennal segments IV (AS IV): the length of antennal segments IV; pronotal length (PL): the length of the pronotum along the midline; pronotal width (PW): the widest width between the pronotal lateral angles; scutellar length (SL): the longest length of scutellum; scutellar width (SW): the widest lateral width of scutellum.

### 2.3. Molecular Study

DNA extraction was performed on 8 *Mictis* specimens. Total genomic DNA was extracted from thoracic and abdominal muscle tissues obtained through a dorsal abdominal incision using TSINGKE Animal DNA Extraction Kit (Universal) (Beijing Tsingke Biotech Co., Ltd., Beijing, China). Fragments of a single mitochondrial DNA (mtDNA) locus, cytochrome oxidase subunit I (COI), were amplified using primers LCO1490 (5′–GGTCAACAAATCATAAAGATATTGG–3′) and HCO2198 (5′–TAAACTTCAGGGTGACCAAAAAATCA–3′) [11]. All DNA extractions, PCR amplifications and sequencing steps were conducted by Beijing Tsingke Biotech Company (Beijing, China). Sequences were deposited in GenBank under accession numbers PV992623–PV992630.

Additional COI data of *Mictis* were downloaded from Genbank, and outgroup selection followed [12], including *Anoplocnemis curvipes*, *Anoplocnemis dallasi* and *Anoplocnemis phasianus*. All sequences were aligned using MEGA 11 [13] with default settings. The final alignment matrix contained 11 sequences of 675 bp. Bayesian inference (BI) analyses were conducted using a partitioned strategy that divided the data into three codon partitions. ModelFinder v2.2.0 [14] was used to select the best-fit partition model (Edge-linked) using the BIC criterion [15]. The estimation and construction of the Bayesian inference (BI) tree were conducted using PhyloSuite v. 1.2.3 [16,17]. The analysis was performed with MrBayes v. 3.2.7a [18] under the GTR + F + G4 model. This involved two independent Markov Chain Monte Carlo (MCMC) runs with default parameters, each consisting of four Metropolis-coupled chains. Each run included 10 million generations, with samples collected every 1000 generations. The initial 25% of samples were discarded as burn-in. Convergence was assessed using Tracer v. 7.1.1 [19], with all parameters achieving an effective sample size (ESS) > 200 and a Potential Scale Reduction Factor (PSRF) close to 1, indicating good convergence [20]. Nodes with Bayesian posterior probabilities (BPP) of 0.95 and above were considered well-supported.

The aligned dataset was also analyzed in a Maximum Likelihood framework using the IQ-TREE (http://iqtree.cibiv.univie.ac.at/, accessed on 9 October 2025) online portal [21]. The best substitution model for each codon partition was chosen using ModelFinder [14], TIM2e + I the best-fit model for the first codon position, HKY + F + I for the second codon position, and HKY + F for the third codon position, and using the Auto parameter with provision for FreeRate heterogeneity. To measure the likelihood of each phylogenetic node in the ML tree, an ultrafast bootstrap (UFBoot) was performed [22], using 1000 iterations to assess clade support. A node was considered robustly supported if UFBoot ≥ 95%. Uncorrected genetic pairwise distances (*p*-distance) were also calculated using MEGA 11 [13].

## 3. Results

### 3.1. Molecular Analyses

**Figure 1:**
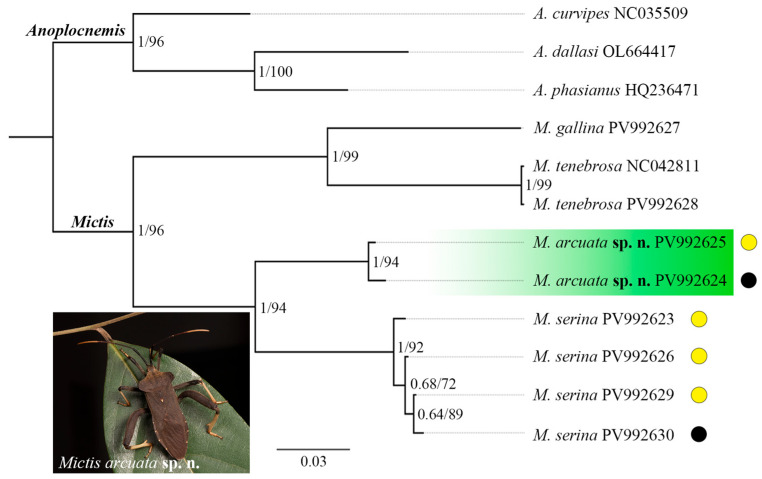
Mitochondrial phylogeny of *Mictis* by Basyesian (BI) analyses and Maximum Likelihood (ML) analyses based on the COI gene. The topology was derived from BI analyses. Numbers at each node indicate Bayesian posterior probabilities and the UFBoot. Yellow balls represent the yellow-tibia type (with pale orange tibiae), and black balls represent the black-tibia type (with dark-brown tibiae). Photo by Han Wan.

DNA barcoding of individuals of *Mictis serina* (yellow tibiae) and *M. fuscipes* (black tibiae) revealed that specimens with black tibiae are nested within the clade formed by those with yellow tibiae (Figure 1). All the individuals together form a monophyletic group, within which the genetic *p*-distance ranges from 0 to 1% (Figure 1; Table 1). This genetic evidence further supports the interpretation that coloration differences reflect intraspecific variation.

DNA barcoding of the new species with varying tibial coloration (pale orange-brown in Lingui, Guangxi, China; dark-brown in Cao Bang, Vietnam) yielded an uncorrected *p*-distance of 0.9% (Figure 1; Table 1).

Phylogenetic analysis reveals that the new species is most closely related to *M. serina*. Despite sharing similar body shapes and tibial color polymorphism, the genetic divergence (*p*-distance 8.2–9.0%; Table 1) is sufficient to differentiate the two species.

### 3.2. Distribution

**Figure 2:**
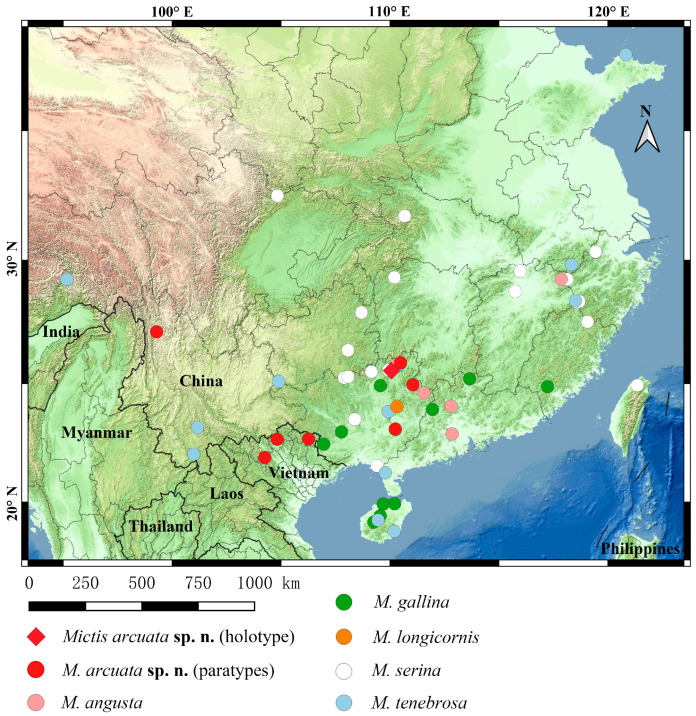
Distribution of *Mictis arcuata* **sp. n.** and distribution of *Mictis* spp. in China.

Most *Mictis* species exhibit remarkably broad distribution ranges, with *M. tenebrosa* spanning from Mêdog, Xizang, in the west to Yantai, Shandong, in the east (Figure 2). The exceptional occurrence of *M. tenebrosa* in Yantai, Shandong, represents the northernmost record of this genus in China and extends far beyond its traditionally recognized range (Figure 2). In addition, all known species of *Mictis* in China, including the new species in this study, can be found in northeastern Guangxi (Figure 2). The new species and *M. serina* also exhibit sympatric distribution.

### 3.3. Taxonomy

**Genus *Mictis* Leach, 1814**

Chinese common name: 侎缘蝽属

(Figure 1, Figure 2, Figure 3, Figure 4, Figure 5, Figure 6, Figure 7, Figure 8, Figure 9, Figure 10, Figure 11, Figure 12, Figure 13, Figure 14 and Figure 15)

*Mictis* Leach, 1814: 91. Type species by monotypy: *Mictis crecifera* Leach, 1814 (=*profana* Fabricius, 1803), Australia.

*Myctis* Leach, 1815: 121 (footnote). For *Mictis* Leach, 1814. Unjustified emendation or error.

*Cerbus* Hahn, 1831: 7, 14 (syn. Westwood, 1842: 4). Type species by subsequent designation (O’Shea & Schaefer, 1980: 229): *Cerbus fulvicornis* sensu Hahn, 1831 (=*longicornis* Westwood, 1842), Oriental Region.

*Mictes* Stål, 1856: 193 (subsequent misspelling).

*Lygaeomictis* Blöte, 1938: 294 (syn. O’Shea & Schaefer, 1980: 229). Type species by original designation: *Lygaeomictis dilatipes* Blöte, 1938, Timor.

Hsiao, 1977: 211–213, keyed six Chinese species.

**Diagnosis.** Body large, stout; head quadrate, preocular pits well developed, antennifers large, prominent, positioned in close proximity, projecting anteriorly beyond the tylus, antennae long, terete; pronotum moderately declivous, pronotum shape variable, posterior margin smooth; scutellum exhibiting faint transverse striations; labium reaching or nearly reaching middle coxae; all femora armed with distal spines, hind femora incrassate in male, dorsal margin smooth or slightly tubercles, ventral margin distinctly tubercles; hind tibiae usually dilated ventrally in male, often bearing a large tooth; common boundary of abdominal segments III and IV forming a usually prominent median tubercle; abdominal segment III with a pair of small to large of ventrolateral tubercles; female seventh sternite featuring an elevated plica forming a blunt angulation; paramere robust, terminating in a short curved apex.

**Distribution.** Oriental, Australian, and Afrotropical regions [5,6].

 

***Mictis arcuata* Xun, Jiang & Zhu sp. n** **.**

**Figure 3:**
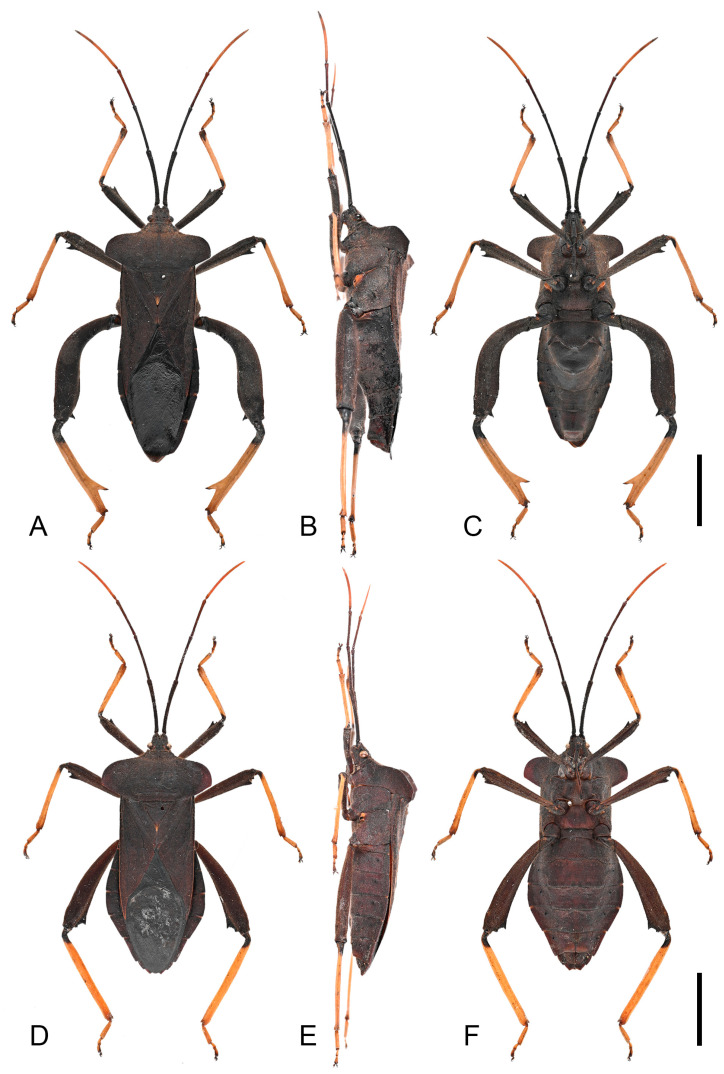
Habitus of *Mictis arcuata* **sp. n.** (**A**–**C**), male, holotype (Lingui, Guangxi, China); (**D**–**F**), female, paratype (Yen Bai, Vietnam); (**A**,**D**), dorsal; (**B**,**E**), lateral; (**C**,**F**), ventral. Scales in 10 mm.

**Figure 4:**
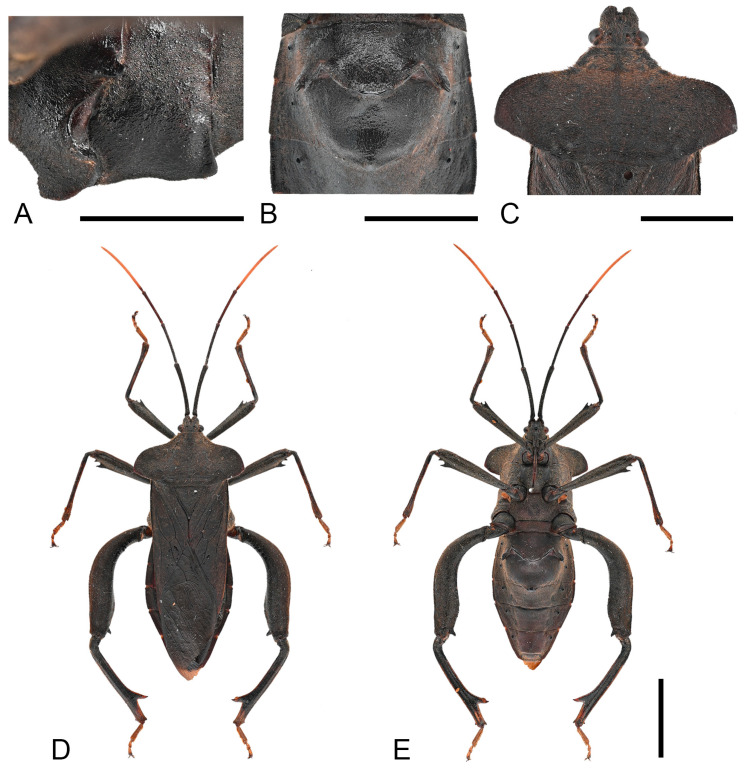
Detailed habitus of *Mictis arcuata* **sp. n.** (**A**–**C**), male, holotype (Lingui, Guangxi, China); (**D**,**E**), male, paratype (Weixi, Yunnan, China), black-tibia type (with dark-brown tibiae); (**A**), abdominal segments III and IV, lateral; (**B**), abdominal segments III–IV, ventral; (**C**), pronotum; (**D**), dorsal; (**E**), ventral. Scales in 10 mm.

**Figure 5:**
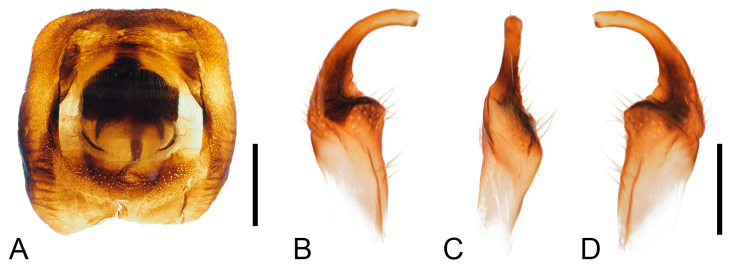
Male genitalia of *Mictis arcuata* **sp. n.**, male, holotype (Lingui, Guangxi, China). (**A**), genital capsule, dorsal; (**B**–**D**), right paramere in their different aspects, photos by Hanqiang Wang. (**A**), scale in 1 mm; (**B**–**D**), scale in 0.5 mm.

Chinese common name: 宽肩侎缘蝽

(Figure 1, Figure 3, Figure 4, Figure 5 and Figure 13A,B,I)

*ZooBankLSID*: urn:lsid:zoobank.org:act: 159D25FD-CB32-484D-8A76-1F789D5FBE32

**Type material. Holotype.** ♂, China, Guangxi Zhuang Autonomous Region, Guilin City, Lingui District, Wantian Yao Ethnic Township, 25.5347° N, 110.0706° E, alt. 220 m, 9-IX-2024, Haofei Fan leg. (IZCAS). **Paratypes.** 1♀, China, Guangxi Zhuang Autonomous Region, Guilin City, Xing’an County, Gaozhai Village, 25.8555° N, 110.4852° E, alt. 492 m, 13-VII-2011, Hanyong Liu leg. (SHEM); 1♂, China, Guangxi Zhuang Autonomous Region, Guiping City, Junfeng Village, 23.0931° N, 110.2453° E, alt. 133 m, VII-2024, De Huang leg. (CHX); 1♂, China, Hunan Province, Yongzhou City, Jiangyong County, Yangjia, 24.9408° N, 111.0570° E, alt. 488 m, 27-IX-2025, Jianping Rong leg. (CHX); 1♂, China, Yunnan Province, Weixi Lisu Autonomous County, Xuelong Mountain, 27.1647° N, 99.3234° E, alt. 2 220 m, X-2023, Guirong Zhang leg. (CHX); 1♂1♀, Vietnam, Cao Bang Province, Cao Bang City, 22.6754° N, 106.2753° E, alt. 208 m, IV-2024, local villagers leg. (SHEM); 1♂, Vietnam, Ha Giang Province, Vi Xuyen district, 22.6631° N, 104.8275° E, alt. 586 m, VII-2025, local villagers leg. (CHX); 1♂1♀, Vietnam, Yen Bai Province, Muong Khuong District, Nam Co Commune, 21.8380° N, 104.2969° E, alt. 943 m, IV-2023, local villagers leg. (CMO, IZCAS).

**Diagnosis.** *Mictis arcuata* **sp. n.** can be diagnosed from all other species by the following morphological characters: (1) body size large (28.2–31.7 mm); (2) dark-brown, integument with irregular, short, reddish-brown serration; (3) pronotum extremely extended laterally beyond lateral margins of corium base; (4) anterolateral borders arcuate, smooth, without serration; (5) humeral angles obtuse; (6) tibiae pale orange with dark-brown base to entirely dark-brown; (7) male hind tibia with a large spine at posterior half; (8) male with entire abdominal segments IV produced into a tubercle; (9) male posterior margin of segment IV slightly produced, overlapping anterior margin of segment V.

**Description. *Color and vestiture*.** Dark-brown; antennal segment III reddish-brown, and segment IV orange-brown; apex of scutellum pale orange; tibiae pale orange with dark-brown base to entirely dark-brown, tarsi pale orange-brown to pale brown (Figure 3, Figure 4 and Figure 13A,B); body covered with short, suberect, reddish-brown setae.

***Head.*** Quadrate, width across eyes greater than length; eyes small, flat; antenniferous tubercle large, prominent; antennae long, terete, relatively stout, relative lengths of antennal segments IV > I > II > III; labium reaching posterior margin of mesosternum.

***Thorax.*** Pronotum steeply declivent, extremely extended, beyond lateral margins of corium base, smooth, without tubercles; collar wide; anterolateral borders arcuate, smooth, without serration; humeral angles obtuse; posterolateral borders sinuate (Figure 4C). Scutellum smooth, with obscure transverse striations; apex flat. Metapleura (both sides) each bearing a large tubercle. Hemelytra reaching the apex of the last abdominal segment. ***Legs.*** Fore and mid femora smooth, slightly incrassate (both sexes), hind femora more incrassate (especially in male), all femora ventrally with two subdistal spines (male hind femur with only one distinct spine); male hind femora distinctly curved, basally with faint tubercles, apical swelling slightly wider than base; female hind femora smooth; male hind tibia with a large spine at posterior half (Figure 13A,B).

***Male genitalia.*** Male genitalia capsule opening nearly square, with small golden setae; ventral rim broadly and shallowly depressed; cuplike sclerite small, broadly rounded. Parameres small, base stout, apical 1/3 evenly curved, with small, narrow, curved tridentate tip; central base to apical part with several large setae; external surface of base with deep, wide, transverse grooves (Figure 5).

**Measurements [**in mm, ♂(*n* = 4)/♀(*n* = 3)**].** BL: 28.2–30.6/28.9–31.7; HL: 1.9–2.2/1.8–2.4; HW: 3.1–3.4/3.2–3.3; IS: 1.1–1.3/1.1–1.2; AS I: 6.4–7.2/6.3–7.3; AS II: 5.5–6.1/4.8–6.9; AS III: 4.5–5.2/4.3–5.3; AS IV: 7.0–7.4/7.0–7.3; PL: 6.4–7.7/5.9–7.7; PW: 12.9–14.1/12.0–14.9; SL: 3.5–4.5/4.0–4.6; SW: 3.8–4.2/4.4–4.5.

**Etymology.** The specific epithet is the Latin adjective arcuatus (-a, -um) referring to the arcuate anterolateral borders of the pronotum.

**Distribution (Figure 2).** China: Guangxi (Guilin, Guiping), Hunan (Jiangyong), Yunnan (Weixi); Vietnam: (Cao Bang, Yen Bai).

**Host plant.** Lauraceae.

**Remarks.** Examination of specimens revealed chromatic variation in tibiae: some individuals exhibit pale orange tibiae with dark-brown base, while others possess entirely dark-brown tibiae, occurring sympatrically (Figure 13A,B). Morphological studies demonstrated no discernible differences beyond tibial coloration, including male genital structures (Figure 3 and Figure 4). Combined with the result of the molecular study, given their high morphological consistency and minimal genetic divergence, we propose these variants as conspecific.

 

***Mictis angusta* Hsiao, 1965**

**Figure 6:**
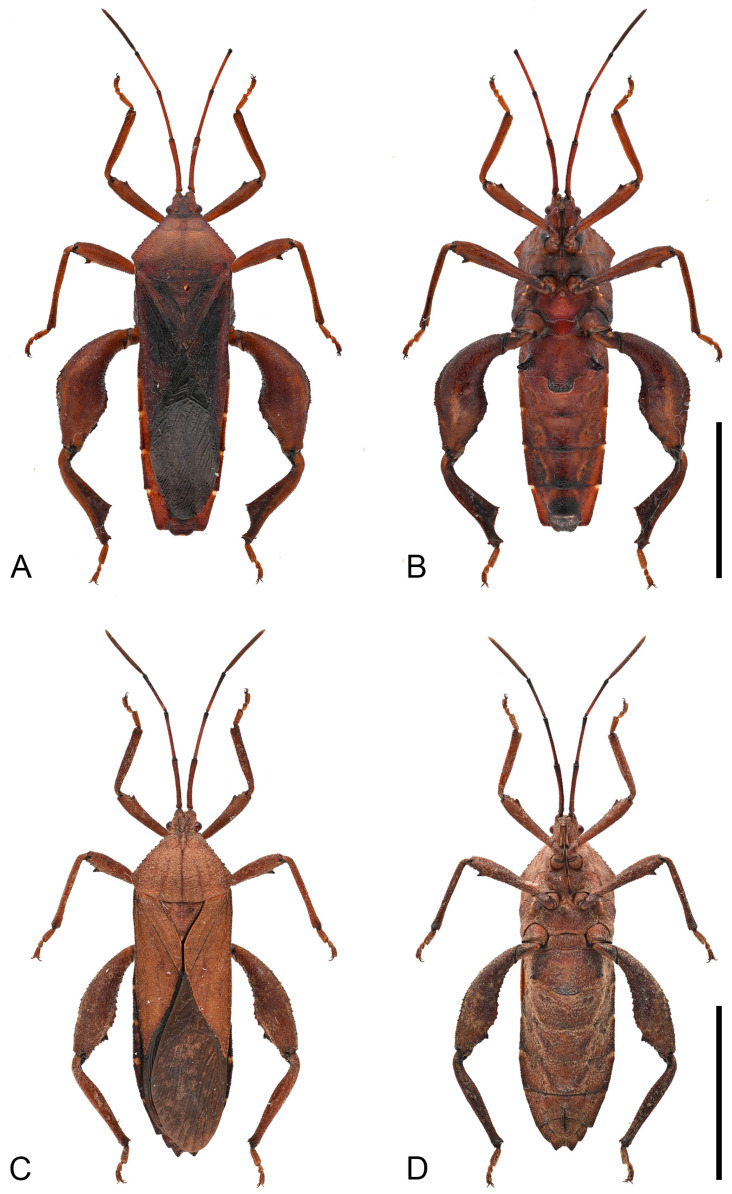
Habitus of *Mictis angusta.* (**A**,**B**), male (Gaoming, Guangdong, China); (**C**,**D**), female, (Qingxin, Guangdong, China); (**A**,**C**), dorsal; (**B**,**D**), ventral. Scales in 10 mm.

Chinese common name: 狭侎缘蝽

(Figure 6 and Figure 13C,J)

*Mictis angusta* Hsiao, 1965: 423, 432. HT: ♂, China, Jiangxi, Wuyuan; NKUM.

**Type material. Holotype.** ♂, China, Jiangxi Province, Wuyuan County, VII-1934, Yu tse Hong leg. (NKUM).

**Additional material examined.** 1♂, China, Guangdong Province, Foshan City, Gaoming District, Hecheng Subdistrict, 22.8983° N, 112.8569° E, alt. 12 m, VII-2023, Hansheng Qu leg. (CHX); 1♀, China, Guangdong Province, Qingyuan City, Qingxin District, Jintan Town, 24.0594° N, 112.8090° E, alt. 66 m, 20-IV-2024, Jiancheng Lei leg. (CHX); 1♀, same locality as above (Qingxin District, Jintan Town), 7-V-2025, Jiancheng Lei leg. (CHX); 2♂1♀, China, Guangxi Zhuang Autonomous Region, Guilin City, Xing’an County, Yong’an Village, 25.7949° N, 110.3056° E, alt. 388 m, 10-VII-2009, Jianhua Huang leg. (SHEM); 1♀, China, Guangxi Zhuang Autonomous Region, Hezhou City, Pingui District, Gupo Mountain, 24.6145° N, 111.5642° E, alt. 676 m, VII-2011, intern students of Guangxi Normal University leg. (SHEM).

**Diagnosis.** *Mictis angusta* can be diagnosed from all other species by the following morphological characters: (1) body size small (19.3–22.2 mm), narrow; (2) chestnut-brown, integument with yellowish-brown setae; (3) humeral angles barely extended, obtuse; (4) anterolateral borders obliquely straight and slightly serrate; (5) male hind tibia with a large leaf-like spine at posterior half; (6) male with common boundary of abdominal segments III and IV produced into a large median tubercle; (7) female abdominal segment III with a pair of small ventrolateral tubercles.

**Measurements [**in mm, ♂(*n* = 4)/♀(*n* = 7)**].** BL: 19.3–22.2/19.4–21.4; HL: 1.2–1.5/1.2–1.4; HW: 2.1–2.6/2.1–2.3; IS: 0.8–0.9/0.8–0.9; AS I: 3.0–3.8/2.7–3.3; AS II: 2.7–3.3/2.5–3.2; AS III: 2.3–2.9/2.2–2.5; AS IV: 2.9–3.7/2.5–3.4; PL: 3.9–5.1/4.6–4.9; PW: 5.6–7.0/6.0–6.9; SL: 2.0–2.4/2.1–2.3; SW: 2.3–2.6/2.3–2.7.

**Distribution (Figure 2).** China: Fujian (Shaowu) [23], Guangdong (Foshan, Qingyuan), Guangxi (Guilin, Hezhou), Jiangxi (Wuyuan), Taiwan [24].

**Remarks.** This species was originally described in 1965 based on a single male specimen, with no female records reported until now [25]. This study documents the female specimens of this species for the first time, revealing several distinctive morphological characteristics in females. Notably, females exhibit a small median tubercle at common boundary of abdominal segments III and IV, along with a pair of small ventrolateral tubercles on abdominal segment III (Figure 6C). These features contrast with most *Mictis* species, where females typically have smooth ventral abdominal surfaces without tubercles.

 

***Mictis gallina* Dallas, 1852**

**Figure 7:**
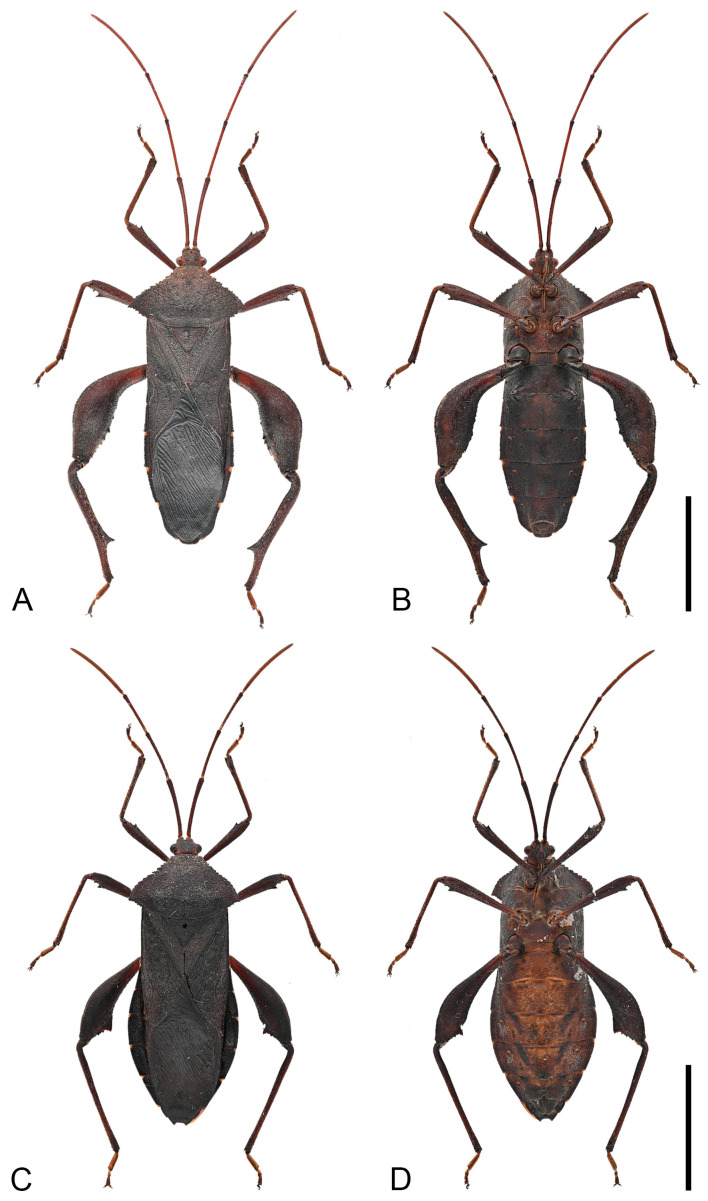
Habitus of *Mictis gallina.* (**A**,**B**), male (Xiuying, Hainan, China); (**C**,**D**), female, (Rongan, Guangxi, China); (**A**,**C**), dorsal; (**B**,**D**), ventral. Scales in 10 mm.

Chinese common name: 锐肩侎缘蝽.

(Figure 7 and Figure 13D,K).

*Mictis gallina* Dallas, 1852: 403. Syntypes: Bangladesh, Sylhet (as Silhet); BMNH.

**Type material. Lectotype** (here designated). ♂, Indian Subcontinent, Bangladesh, Sylhet (as Silhet), 23.5000° N, 91.6667° E (BMNH 884212). **Paralectotype** (here designated). ♀, same data as lectotype (BMNH 884213).

**Additional material examined.** 2♀, China, Fujian Province, Zhangzhou City, Nanjing County, Hexi Town, 24.8763° N, 117.2321° E, alt. 220 m, 12-V-1962, Yongcheng Lin leg. (SHEM); 1♂, China, Guangdong Province, Shaoguan City, Renhua County, Jimageng Village, 25.1969° N, 113.6591° E, alt. 505 m, 11-V-2025, Han Wan leg. (CHX); 1♂, China, Guangdong Province, Zhaoqing City, Fengkai County, Yidou Mountain, 23.9269° N, 111.9531° E, alt. 537 m, 22-V-2023, Hengjing Liang leg. (CZZ); 1♂1♀, China, Guangxi Zhuang Autonomous Region, Liuzhou City, Rongan County, Qiaoban Village, 24.9172° N, 109.5622° E, alt. 456 m, 20-V-2023, Changlai Luo leg. (CHX); 1♂1♀, China, Guangxi Zhuang Autonomous Region, Chongzuo City, Longzhou County, Nonggang, 22.4679° N, 106.9608° E, alt. 261 m, 18–23-VII-1995, Xianwei Liu, Weinian Zhang & Xingbao Jin leg. (SHEM); 4♂4♀, same locality as above (Longzhou County, Nonggang), 11–15-VII-2013, Weibing Zhu & Haiguang Zhang leg. (SHEM); 1♂1♀, China, Guangxi Zhuang Autonomous Region, Nanning City, Long’an County, Gutan Village, 22.9830° N, 107.7772° E, alt. 99 m, 17-X-2024, Yunneng Fan leg. (CHX); 2♀, China, Hainan Province, Baisha Li Autonomous County, Bawangling National Nature Reserve, 19.1346° N, 109.2619° E, alt. 780 m, 24-IV-2017, Xianwei Liu leg. (SHEM); 1♀, China, Hainan Province, Danzhou City, Nanfeng Town, 7-VII-1932, F. K. To leg. (NKUM); 1♂, China, Hainan Province, Haikou City, Xiuying District, Shishan Town, Leiqiong Haikou Volcanic Geopark, 19.9254° N, 110.2140° E, alt. 191 m, 2-VII-2022, Michael Zelun Lee leg. (CHX); 1♂, China, Hainan Province, Lingao City, 23–25-V-1932, F. K. To leg. (NKUM).

**Diagnosis.** *Mictis gallina* can be diagnosed from all other species by the following morphological characters: (1) body size medium (21.3–26.6 mm); (2) reddish-brown to dark-brown; (3) humeral angles extended, pointed, with serrate margins; (4) anterolateral borders obliquely straight and distinct serrate; (5) male hind tibia with a large spine at posterior half; (6) male hind femora serrate along ventral margin; (7) male without median abdominal tubercle, only ventrolateral tubercles present.

**Measurements [**in mm, ♂(*n* = 3)/♀(*n* = 4)**].** BL: 22.4–26.6/21.3–23.2; HL: 1.3–1.7/1.3–1.5; HW: 2.5–2.8/2.5–2.7; IS: 1.0–1.1/0.9–1.0; AS I: 5.3–6.2/4.9–5.2; AS II: 4.2–5.2/4.1–4.6; AS III: 3.6–4.4/3.2–4.0; AS IV: 5.4–7.1/5.5–6.2; PL: 5.4–6.9/5.3–5.8; PW: 8.1–10.5/7.8–8.7; SL: 2.2–2.6/2.3–2.8; SW: 2.8–3.2/3.1–3.5.

**Distribution (Figure 2).** China: Fujian (Zhangzhou), Guangdong (Meizhou, Zhaoqing) [23], Guangxi (Liuzhou, Chongzuo, Nanning), Hainan (Baisha, Danzhou, Haikou, Lingao), Hong Kong [26], Jiangxi [23], Yunnan (Hekou) [23]; Bangladesh; India [2]; Myanmar [2].

 

***Mictis longicornis* Westwood, 1842**

**Figure 8:**
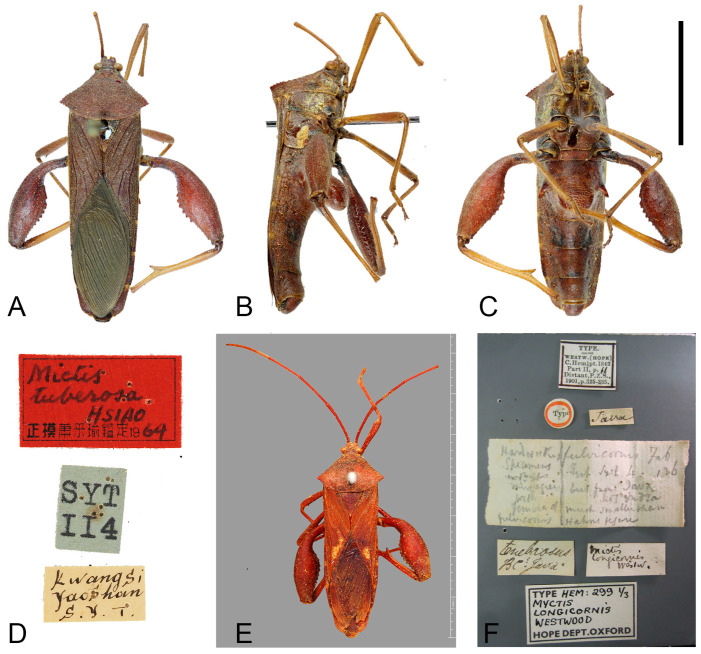
Type specimens of *Mictis* species. (**A**–**D**) *Mictis tuberosa*, male, holotype (Yaoshan, Guangxi, China); (**E**,**F**) *Mictis longicornis*, male, lectotype (Jawa); (**A**,**E**) dorsal; (**B**) lateral; (**C**) ventral; (**D**,**F**) labels. The Chinese text in (**D**) indicates ‘holotype, identified by Tsai-Yu Hsiao’. (**A**–**C**) scale in 10 mm; (**E**) scale in 1 mm. (**A**–**D**) photos by Hanqiang Wang, ©NKUM; (**E**,**F**) photos by Laurence Livermore, from Coreoidea Species File Version 5.0/5.0, published under CC BY-SA 4.0 International Licence, ©UMO.

Chinese common name: 长角侎缘蝽

(Figure 8 and Figure 13E,L)

*Myctis longicornis* Westwood, 1842: 4, 11–12. Syntypes: Jawa; UMO. *Mictis longicornis*, Stål, 1873: 45 (cataloged).

*Mictis conjunctus* Herrich-Schäffer, 1850: 247 (Syn. Stål, 1873: 45). Syntype: without locality; lost.

*Mictis tuberosa* Hsiao, 1965: 423, 432. HT: ♂, China, Guangxi, Yaoshan; NKUM. Hsiao, 1977: 212 (listed); O’Shea & Schaefer, 1980: 231 (listed); Dolling, 2006: 93 (listed). **Syn. n.**

**Type material.** *Mictis longicornis* Westwood, 1842. **Lectotype** (here designated). ♂, Jawa (UMO HEMI 0299.1). **Paralectotype** (here designated). ♀, same data as lectotype (UMO HEMI 0299.2).

*Mictis tuberosa* Hsiao, 1965. **Holotype.** 1♂, China, Guangxi Zhuang Autonomous Region, Yaoshan County (NKUM).

**Additional material examined.** 1♂1♀, Malaysia, Sabah, Ranau District, Poring Hot Spring, 6.0568° N, 116.7055° E, alt. 553 m, 21-II-2024, Haoran Gao & Zixu Yin leg. (CHX).

**Diagnosis.** *Mictis longicornis* can be diagnosed from all other species by the following morphological characters: (1) body size medium; (2) reddish-brown, integument with yellowish-brown setae; (3) humeral angles extended, pointed, with serrate margins; (4) anterolateral borders obliquely straight with distinct serration; (5) male hind tibia with a large spine at posterior half; (6) male hind femora serrate along ventral margin; (7) male with common boundary of abdominal segments III and IV produced into a large thumb-like median tubercle.

**Distribution (Figure 2).** China: Guangxi (Yaoshan); Indonesia [2]; Malaysia [2], Philippines [2].

**Remarks.** Original description of *Mictis tuberosa* compared it only with *M. gallina*, with no reference to *M. longicornis* [25]. Our examination of the holotypes of *M. tuberosa* and *M. longicornis* confirms their morphological identity: diagnostic traits such as hind femoral and median abdominal tubercles are entirely congruent (Figure 8). However, *M. longicornis* is restricted to the Malay Peninsula and Southeast Asian islands, while *M. tuberosa* was purportedly collected in northern Indochina. Notably, no subsequent records of *M. tuberosa* have been confirmed from this region since its original description in 1965, raising questions about the accuracy of its reported collection site. This discrepancy necessitates verification through targeted fieldwork in Indochina. Given currently no difference in potential specific importance, we formally relegate *Mictis tuberosa* to a subjective junior synonym of *Mictis longicornis*.

 

***Mictis serina* Dallas, 1852**

**Figure 9:**
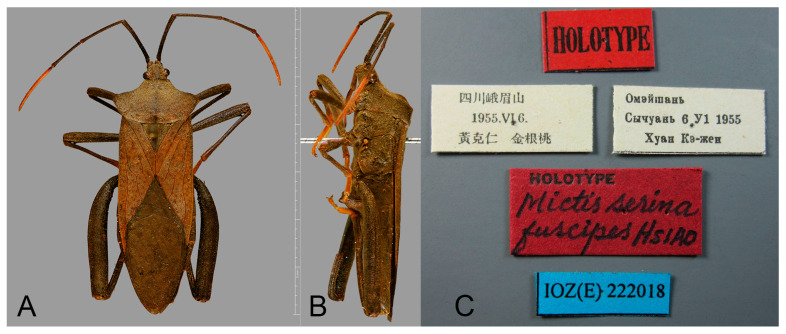
Holotype of *Mictis fuscipes*. (**A**–**C**), male, holotype (Emei, Sichuan, China); (**A**), dorsal; (**B**), lateral; (**C**), labels, The non-English term in (**C**) indicates collection details: Mont. Emei, 6-VI-1955, Keren Huang & Gentao Jin. Scale in mm. Photos by Laurence Livermore, from Coreoidea Species File Version 5.0/5.0, published under CC BY-SA 4.0 International Licence, ©IZCAS.

**Figure 10:**
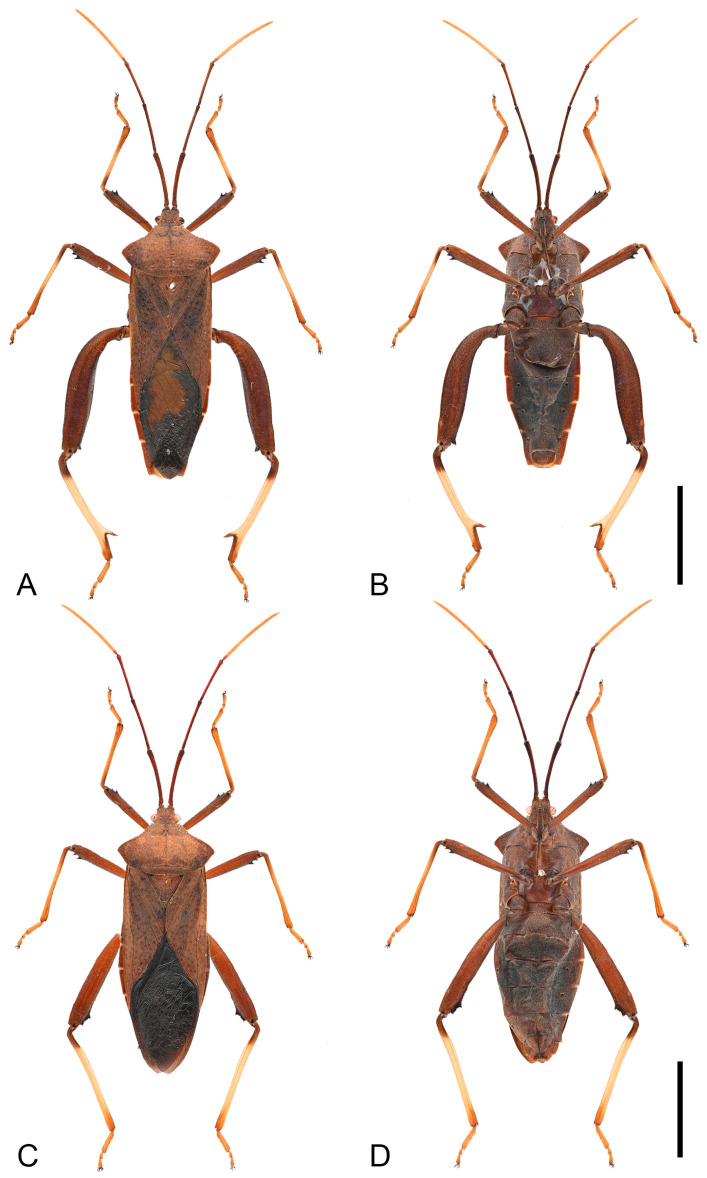
Habitus of *Mictis serina.* (**A**,**B**), male (Tieshangang, Guangxi, China); (**C**,**D**), female, (Tieshangang, Guangxi, China); (**A**,**C**), dorsal; (**B**,**D**), ventral. Scales in 10 mm.

**Figure 11:**
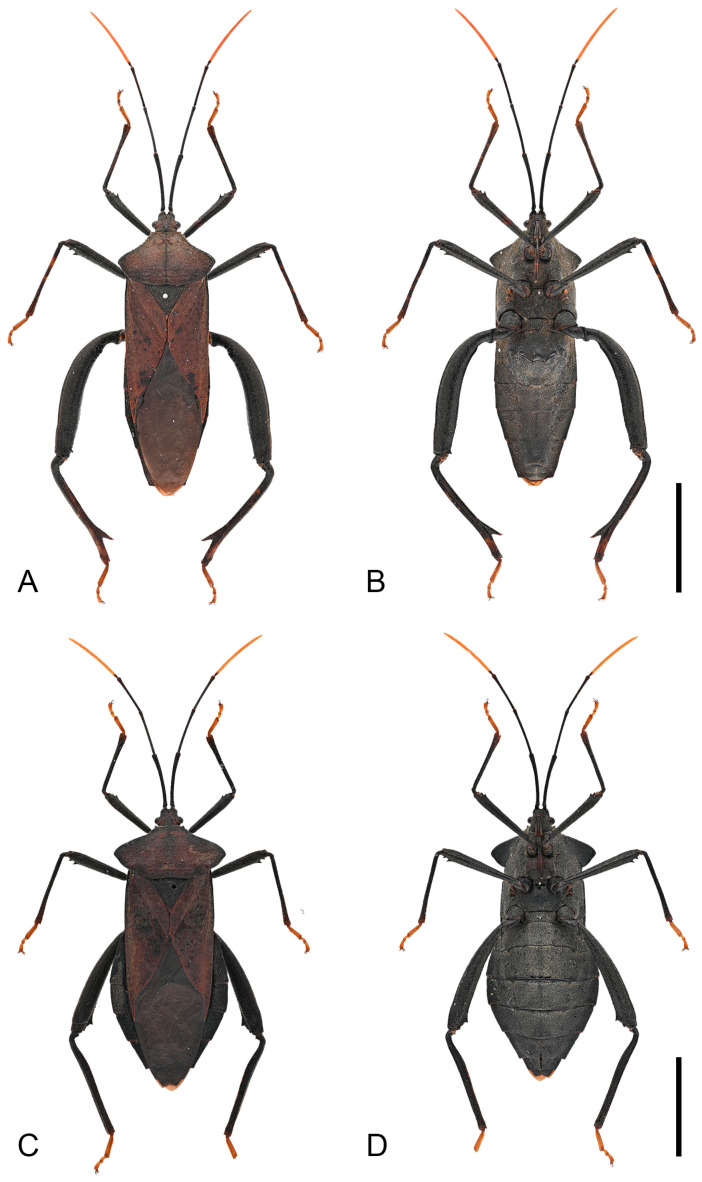
Habitus of *Mictis serina*, black-tibia type (with dark-brown tibiae). (**A**,**B**), male (Tieshangang, Guangxi, China); (**C**,**D**), female, (Tieshangang, Guangxi, China); (**A**,**C**), dorsal; (**B**,**D**), ventral. Scales in 10 mm.

Chinese common name: 黄胫侎缘蝽

(Figure 9, Figure 10, Figure 11 and Figure 13F,G,M)

*Mictis serina* Dallas, 1852: 403. Syntype: ♀, China; BMNH.

*Mictis serina fuscipes* Hsiao, 1963: 311, 337 (as color variety of *Mictis serina*; subspecies upgraded to species by Hsiao, 1964: 9, 17); O’Shea & Schaefer, 1980: 231 (listed). HT: ♂, Sichuan, Mt Emei; IZACS. *Mictis fuscipes*, Hsiao, 1977: 212 (listed); Dolling, 2006: 92, 93 (listed). **Syn. n.**

**Type material.** *Mictis serina* Dallas, 1852. **Lectotype** (here designated). ♀, China (BMNH 884224).

*Mictis fuscipes* Hsiao, 1963. **Holotype.** ♂, China, Sichuan, Mt Emei (IZCAS 222018).

**Additional material examined.** 1♂1♀, China, Guangxi Zhuang Autonomous Region, Beihai City, Tieshangang District, Longmen Village, 21.5478° N, 109.4026° E, alt. 27 m, 15-VI-2023, Jianxie Liu leg. (CHX); 2♂, China, Guangxi Zhuang Autonomous Region, Chongzuo City, Longzhou County, Nonggang, 22.4679° N, 106.9608° E, alt. 261 m, 18–23-VII-1995, Xianwei Liu, Weinian Zhang & Xingbao Jin leg. (SHEM); 5♂1♀, same locality as above (Longzhou County, Nonggang), 13-VII-2013, Weibing Zhu & Haiguang Zhang leg. (SHEM); 3♂, China, Guangxi Zhuang Autonomous Region, Guiping City, Junfeng Village, 23.0931° N, 110.2453° E, alt. 133 m, VII-2024, De Huang leg. (CHX); 1♀, China, Guangxi Zhuang Autonomous Region, Guilin City, Lingui District, Anjiangping, 25.5614° N, 109.9455° E, alt. 1314 m, 25-V-2023, Feiyu Duan leg. (CHX); 2♀, China, Guangxi Zhuang Autonomous Region, Guilin City, Xing’an County, Gaozhai Village, 25.8555° N, 110.4852° E, alt. 492 m, 13-VII-2011, Hanyong Liu leg. (SHEM); 1♂, China, Guangxi Zhuang Autonomous Region, Hezhou City, Pingui District, Gupo Mountain, 24.6145° N, 111.5642° E, alt. 676 m, 4-VII-2009, Jianhua Huang leg. (SHEM); 1♂1♀, China, Guangxi Zhuang Autonomous Region, Liuzhou City, Rongshui Miao Autonomous County, Hongshui Township, Liangshuang Village, 25.4928° N, 109.1519° E, alt. 570 m, 7-VI-2023, local villagers leg. (CHX); 1♀, China, Guangxi Zhuang Autonomous Region, Nanning City, Wuming District, Damingshan Mountain, 23.5145° N, 108.3879° E, alt. 850 m, VII–IX-2012, collector unknown (SHEM); 2♂, China, Guizhou Province, Leishan County, Wukai Village, 26.3784° N, 108.0780° E, alt. 858 m, 7-VII-1988, Zuyao Liu leg. (SHEM); 3♀, China, Guizhou Province, Libo County, Maolan National Nature Reserve, Weng’ang Township, 25.2474° N, 107.9053° E, alt. 851 m, 24–26-VII-2015, Weibing Zhu leg. (SHEM); 3♂2♀, China, Guizhou Province, Tongren City, Jiangkou County, Fanjing Mountain, 27.9136° N, 108.6941° E, alt. 1 701 m, 12–15-VII-1988, Zuyao Liu leg. (SHEM); 1♂1♀, China, Hubei Province, Shennongjia Forestry District, Yangri Town, 31.7446° N, 110.6760° E, alt. 986 m, 14–15-VII-1983, Gentao Jin & Zuyao Liu leg. (SHEM); 1♂1♀, China, Hunan Province, Zhangjiajie City, Zhushitou Forest Farm, 29.3214° N, 110.2039° E, alt. 995 m, 13-VI-1988, Zuyao Liu leg. (SHEM); 1♀, China, Jiangxi Province, Jiujiang City, Lushan District, Guling Town, 17-VIII-1935, O. Piel leg. (NKUM); 1♂1♀, China, Jiangxi Province, Nanchang City, Xinjian District, Meiling National Forest Park, Yueliangwan, 28.7601° N, 115.7529° E, alt. 146 m, 9-V-2023, Tianxiao Feng leg. (CZZ); 1♂, China, Taiwan Province, New Taipei City, Sanxia District, 24.9318° N, 121.3575° E, alt. 92 m, 23-V-2025, Zhenfeng Jian leg. (CHX); 1♂, China, Zhejiang Province, Hangzhou City, Lin’an District, Tianmu Mountain, 25-VII-1936, collector unknown (NKUM); 1♂, same locality as above (Lin’an District, Tianmu Mountain), 3-VIII-1962, Zhizi Chen leg. (SHEM); 1♀, same locality as above (Lin’an District, Tianmu Mountain), 14-VIII-1962, Gentao Jin leg. (SHEM); 1♂, China, Zhejiang Province, Lishui City, Qingyuan County, Wuligen, 27.5360° N, 119.0685° E, alt. 643 m, 12–20-VIII-1996, Weinian Zhang & Xingbao Jin leg. (SHEM); 1♂, China, Zhejiang Province, Jiangshan City, Shuangxikou Town, Laofoyan, 28.3619° N, 118.6889° E, alt. 643 m, 4-VII-2017, Hanqiang Wang leg. (SHEM); 1♂, China, Zhejiang Province, Jiangshan City, Zhou Village, 28.3264° N, 118.6175° E, alt. 643 m, 11-VIII-2016, Damin Zhang, Weibing Zhu & Xianwei Liu leg. (SHEM); 1♀, China, Zhejiang Province, Quzhou City, Kaihua County, Gutian Mountain, 29.2456° N, 118.1345° E, alt. 502 m, 15–17-VII-2012, Hanqiang Wang, Li Dai & Xianwei Liu leg. (SHEM); 1♂, China, Sichuan, Guangyuan City, Qingcuan County, Tangjiahe National Nature Reserve, Gongnong Village, 32.5282° N, 104.8342° E, alt. 1 112 m, 25-VII-2024, Zixu Yin leg. (EANU).

**Diagnosis.** *Mictis serina* can be diagnosed from all other species by the following morphological characters: (1) body size large (19.8–28.1 mm); (2) reddish-brown to dark-brown, integument with yellowish-brown setae; (3) pronotum slightly expanded beyond base of corium; (4) anterolateral borders curved, smooth, barely serrate; (5) humeral angles obtuse; (6) tibiae pale orange with dark-brown base to entirely dark-brown; (7) male hind tibia with a large spine at posterior half; (8) male with common boundary of abdominal segments III and IV produced into a large thumb-like median tubercle.

**Measurements [**in mm, ♂(*n* = 8)/♀(*n* = 8)**].** BL: 19.8–27.4/23.4–28.1; HL: 1.3–2.1/1.3–1.8; HW: 2.5–2.8/2.6–2.8; IS: 0.8–1.0/0.9–1.0; AS I: 4.2–5.9/4.5–5.4; AS II: 3.9–5.0/3.4–5.0; AS III: 3.4–4.5/3.3–4.2; AS IV: 5.5–7.1/5.7–6.7; PL: 4.1–5.9/5.3–6.1; PW: 7.0–9.6/8.9–11.1; SL: 2.4–3.2/2.9–3.8; SW: 2.6–4.0/3.3–4.0.

**Distribution (Figure 2).** China: Fujian (Fuzhou, Shaowu) [23], Guangdong (Meizhou, Qingyuan, Zhaoqing) [23], Guangxi (Beihai, Chongzuo, Guiping, Guilin, Hezhou, Liuzhou, Nanning), Guizhou (Leishan, Libo, Tongren), Hong Kong [26], Hubei (Shennongjia), Hunan (Zhangjiajie), Jiangxi (Jiujiang, Wuyuan) [23], Sichuan (Emeishan, Guangyuan, Yaan) [23], Taiwan, Zhejiang (Hangzhou, Lishui, Jiangshan, Quzhou).

**Remarks.** Hsiao, in 1963, established the subspecies *Mictis serina fuscipes* based on comparison with *Mictis serina*, distinguishing it by its slightly darker body coloration, uniformly black tibiae, and orange tarsi (Figure 9) [27]. In 1964, he elevated this subspecies to species rank as *Mictis fuscipes*, primarily based on tibial coloration [28]. However, after examining a large number of specimens, we found that tibial color exhibits continuous variation. Individuals with different tibial coloration can be collected from the same locality, and apart from tibial color, all other morphological features are identical (Figure 10, Figure 11 and Figure 13F,G). These findings suggest that body and tibial coloration are more likely to represent individual variation rather than interspecific differences. According to the results of DNA barcode research, the genetic evidence further supports the interpretation that coloration differences reflect intraspecific variation. Based on both morphological and molecular evidence, we formally relegate *Mictis fuscipes* to a subjective junior synonym of *Mictis serina*.

 

***Mictis tenebrosa* Fabricius, 1787**

**Figure 12:**
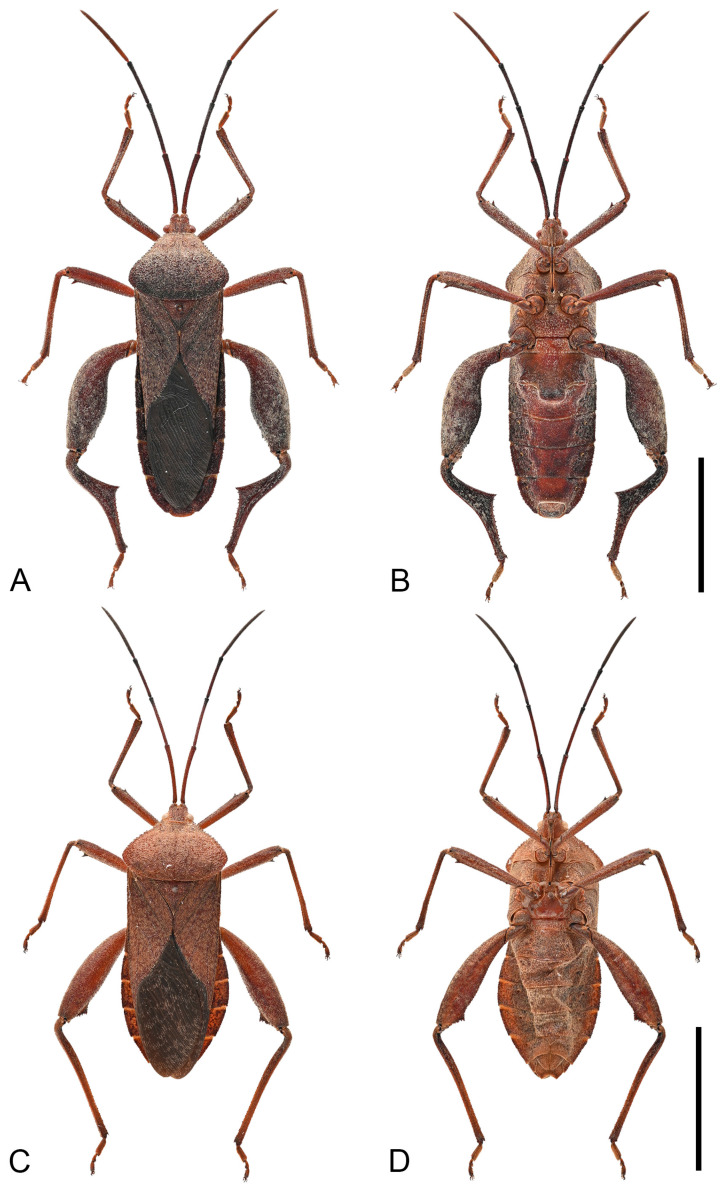
Habitus of *Mictis tenebrosa.* (**A**,**B**), male (Jinghong, Yunnan, China); (**C**,**D**), female, (Suixi, Guangdong, China); (**A**,**C**), dorsal; (**B**,**D**), ventral. Scales in 10 mm.

**Figure 13:**
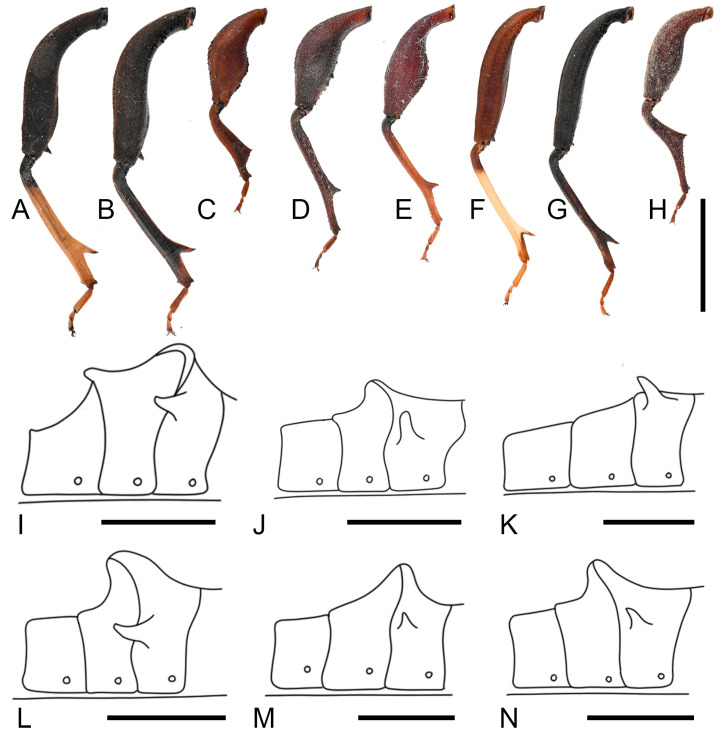
Morphological features of *Mictis* spp., males. (**A**–**H**), left hind tibia; (**I**–**N**), line drawing of abdominal segments III–V, lateral; (**A**,**I**), *M*. *arcuata* **sp. n.**; (**B**), *M*. *arcuata* **sp. n.**, black-tibia type (with dark-brown tibiae); (**C**,**J**), *M*. *angusta*; (**D**,**K**), *M*. *gallina*; (**E**,**L**), *M*. *longicornis*; (**F**,**M**), *M*. *serina*; (**G**), *M*. *serina*, black-tibia type; (**H**,**N**), *M. tenebrosa*. Scales in 10 mm.

Chinese common name: 曲胫侎缘蝽

(Figure 12 and Figure 13H,N)

*Cimex tenebrosus* Fabricius, 1787: 288. Syntypes: E India; BMNH, ZMUC. *Coreus tenebrosus*, Fallén, 1814: 7 (generic placement). *Myctis tenebrosus*, Billberg, 1820: 69 (generic placement). *Anisoscelis tenebrosus*, Latreille, 1829: 197 (generic placement); *Mictis tenebrosus*, Amyot & Serville, 1843: 190 (generic placement).

*Coreus* (*Cerbus*) *umbilicatus* Burmeister, 1834: 296 (Syn. Stål, 1873: 45). Syntype: multiple unsexed adults; ZMHB. *Cerbus umbilicatus*, Burmeister, 1835: 341 (generic placement). *Mictis umbilicatus*, Herrich-Schäffer, 1853:121, 128 (generic placement).

*Mycis fasciatus* Westwood, 1842: 11 (Syn. Stål, 1873: 47, suspected; Distant, 1901: 327). Syntypes: ♂, ♀, China; UMO. *Mictis fasciatus*, Dallas, 1852: 404 (cataloged). *Mictis fasciata*, Dohrn, 1859: 24 (cataloged).

*Mictis nigricornis* Dallas, 1852: 400 (Syn. Stål, 1873: 47, suspected; Distant, 1902: 344). Syntypes: ♀, Bangladesh, Silhet [=Sylhet]; BMNH.

**Type material. Lectotype** (here designated). ♂, Indian Subcontinent, India, East India (ZMUC 00101285). **Paralectotypes** (here designated). 1 unsexed adult, same data as lectotype (ZMUC 00101286); unsexed adult more than 1, same data as lectotype (BMNH).

**Additional material examined.** 1♂1♀, China, Anhui Province, Huangshan City, Tunxi District, 12-VIII-1985, Zuyao Liu leg. (SHEM); 1♂1♀, China, Guangdong Province, Zhanjiang City, Suixi County, Gangmen Town, 21.2518° N, 109.7904° E, alt. 25 m, 30-V-2023, Dexi Kong leg. (CHX); 3♀, China, Guangxi Zhuang Autonomous Region, Guilin City, Xing’an County, Gaozhai Village, 25.8555° N, 110.4852° E, alt. 492 m, 13-VII-2011, Hanyong Liu leg. (SHEM); 2♂4♀, China, Guangxi Zhuang Autonomous Region, Guilin City, Xing’an County, Tongren Village, 25.7952° N, 110.4579° E, alt. 327 m, 2-VII-2006, Yousong Zhang & Zhongcheng Pan leg. (SHEM); 1♀, China, Guangxi Zhuang Autonomous Region, Hezhou City, Pingui District, Gupo Mountain, 24.6145° N, 111.5642° E, alt. 676 m, 6-VII-2009, Jianhua Huang leg. (SHEM); 2♂, China, Guangxi Zhuang Autonomous Region, Laibin City, Jinxiu Yao Autonomous County, Hualu Village, 25-IV-1982, Zuyao Liu & Xingbao Jin leg. (SHEM); 1♂, China, Guizhou Province, Xingyi City, 11-VIII-1958, Daoying Bi & Zhizi Chen leg. (SHEM); 1♂, China, Hainan Province, Baisha Li Autonomous County, 24-III-1959, Gentao Jin leg. (SHEM); 3♂, China, Hainan Province, Wanning City, Xinglong Town, 14-X-1957, Zhizi Chen leg. (SHEM); 1♂1♀, China, Shandong Province, Yantai City, Penglai District, 26-VII-1983, Daoying Bi leg. (SHEM); 1♂, China, Xizang Autonomous Region, Mêdog County, Beibeng Town, 28-VII-1977, Jianyi Wu leg. (SHEM); 1♂, China, Yunnan Province, Puer City, Mohei Town, 6-IV-1982, Gentao Jin leg. (SHEM); 1♂, China, Yunnan Province, Xishuangbanna Dai Autonomous Prefecture, Jinghong City, Jinuo Mountain, Jinuo Ethnic Township, 22.0406° N, 100.9900° E, alt. 1 073 m, 24-III-2023, Haoran Gao leg. (CHX); 3♂2♀, China, Zhejiang Province, Quzhou City, Jiangshan City, Xianxialing, 11-V-1984, Zuyao Liu & Shensheng Zheng leg. (SHEM).

**Diagnosis.** *Mictis tenebrosa* can be diagnosed from all other species by the following morphological characters: (1) body size medium (16.7–22.7 mm); (2) chestnut-brown, integument densely covered with creamy-white setae; (3) humeral angles barely extended, obtuse; (4) anterolateral borders obliquely straight and slightly serrate; (5) male hind tibia with a large leaf-like spine at midpoint; (6) male with common boundary of abdominal segments III and IV produced into a large median tubercle.

**Measurements [**in mm, ♂(*n* = 9)/♀(*n* = 5)**].** BL: 16.7–22.7/20.1–21.3; HL: 1.2–1.7/1.3–1.6; HW: 2.1–2.5/2.3–2.4; IS: 0.8–1.0/0.8–1.0; AS I: 2.7–4.9/3.5–4.4; AS II: 3.1–4.3/3.1–4.0; AS III: 2.4–3.8/2.6–3.6; AS IV: 3.6–5.3/4.0–5.1; PL: 4.1–5.8/5.1–5.6; PW: 5.9–8.2/7.6–8.1; SL: 2.2–3.0/2.3–2.8; SW: 2.4–3.3/2.6–3.2.

**Distribution (Figure 2).** China: Anhui (Huangshan), Fujian (Fuzhou, Jianou) [23], Guangdong (Xinyi, Zhanjiang) [23], Guangxi (Guilin, Hezhou, Laibin, Liuzhou, Yulin) [23], Guizhou (Xingyi), Hainan (Baisha), Hong Kong [26], Hunan (Xiangtan) [23], Jiangxi (Guixi, Shangrao) [23], Shandong (Yantai), Sichuan (Emeishan) [23], Xizang (Mêdog), Yunnan (Jinghong, Puer), Zhejiang (Quzhou, Shaoxing, Zhoushan) [23]; India [2]; Indonesia [2]; Malaysia [2].

**Key to the genus *Mictis* from China**
1.Humeral angles distinctly extended, male hind tibial spine large and spine-like…….2-Humeral angles barely extended, male hind tibial spine small, angulate......................52.Humeral angles extended pointed, with serrate margins…….........................................3-Humeral angles extended rounded…….............................................................................43.Apex of antennal segment IV pale, male with common boundary of abdominal  segments III and IV produced into a large median tubercle.................*Mictis longicornis*-Antennal segment IV uniformly colored, male without median abdominal tubercle,  only ventrolateral tubercles present................................................................*Mictis gallina*4.Pronotum extremely extended laterally, the lateral extension beyond the margins of  the corium base greater than head width; male with entire abdominal segment IV  produced into a large median tubercle...............................................*Mictis arcuata*
**sp. n.**-Pronotum moderately extended laterally, the lateral extension beyond the margins of the corium base less than head width; male with common boundary of abdominal  segments III and IV not produced into a large median tubercle..................*Mictis serina*5.Body narrow, antennae about 3/5 of body length, male hind tibial spine inserted at  2/3 of tibia........................................................................................................*Mictis angusta*-Body moderately broad, antennae about 4/5 of body length, male hind tibial spine  inserted at midpoint of tibia........................................................................*Mictis tenebrosa*


 

***Ochrochira falloui* Reuter, 1888, comb. n.**

**Figure 14:**
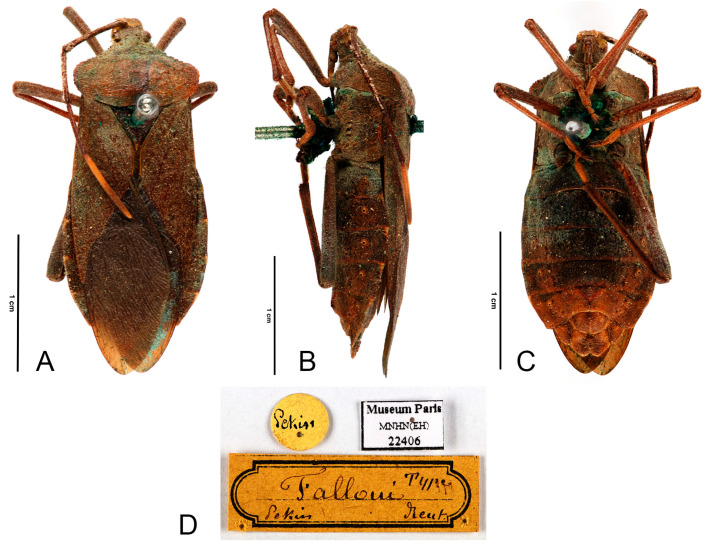
Type specimen of *Ochrochira falloui* **comb. n.** (**A**–**D**), female, lectotype (Beijing, China); (**A**), dorsal; (**B**), lateral; (**C**), ventral; (**D**), labels. Scales in 10 mm. Photos from [29], published under CC BY-NC-ND 4.0 International Licence, ©MNHN.

**Figure 15:**
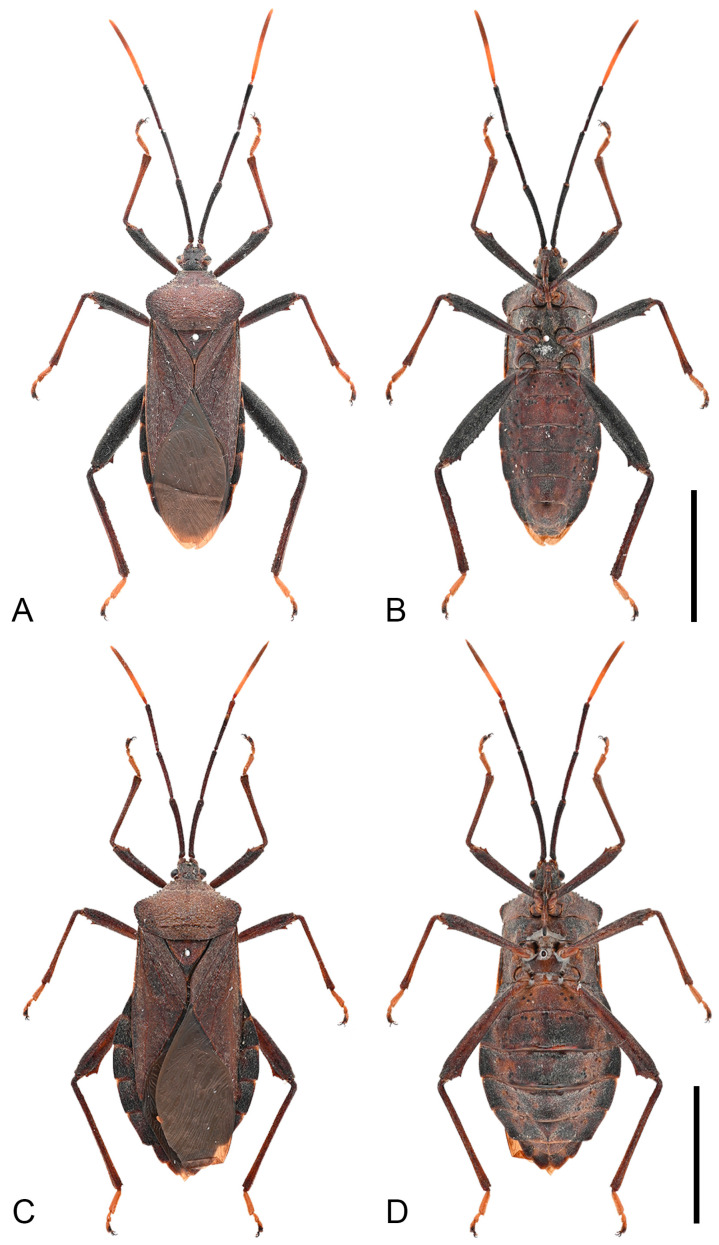
Habitus of *Ochrochira falloui* **comb. n.** (**A**,**B**), male (Changping, Beijing, China); (**C**,**D**), female, (Fangshan, Beijing, China); (**A**,**C**), dorsal; (**B**,**D**), ventral. Scales in 10 mm.

Chinese common name: 北京赭缘蝽

(Figure 14 and Figure 15)

*Mictis falloui* Reuter, 1888: 65. Syntypes: Beijing (as Pekin); MNHN. Lethierry & Se-verin, 1894: 9 (cataloged); Oshanin, 1906: 177 (cataloged); Oshanin, 1912: 20 (cataloged); Kiritshenko, 1916: 48, 57–59 (cataloged; description); Hsiao, 1964 (noted); O’Shea & Schaefer, 1980: 231 (listed); Dolling, 2006: 92 (listed).

**Type material. Lectotype** (here designated). ♀, China, Beijing (as Pekin), 39.9000° N, 116.3830° E (MNHN-EH-EH22406).

**Additional material examined.** 1♂, China, Beijing, Changping District, Nankou Town, Huyu Natural Scenic Area, 40.2813° N, 116.1320° E, alt. 646 m, 1-VI-2023, Michael Zelun Lee leg. (CHX); 2♀, China, Beijing, Fangshan District, Puwa Township, 37.7838° N, 115.5374° E, alt. 1 180 m, 11-VI-2023, Michael Zelun Lee leg. (CHX); 1♀, China, Beijing, Mentougou District, Qingshui Town, Baihua Mountain, 39.8395° N, 115.5786° E, alt. 550 m, 23-VI-2024, Yan Liu leg. (CHX).

**Diagnosis.** *Ochrochira falloui* can be diagnosed from all other species by the following morphological characters: (1) body size medium (19.4–21.2 mm); (2) dark-brown, antennal segment IV yellowish-brown with a dark stripe near the distal end; (3) humeral angles barely extended, obtuse; (4) anterolateral borders slightly serrate; (5) female hind femora smooth, without tubercles; (6) male hind femora incrassate, with tubercles on anterior and posterior surfaces; (7) abdominal dorsum entirely black; (8) abdominal segments III with six black spots; (9) abdomen smooth, without tubercles.

**Measurements [**in mm, ♂(*n* = 1)/♀(*n* = 1)**].** BL: 19.4/21.2; HL: 1.9/1.9; HW: 2.5/2.6; IS: 0.7/0.7; AS I: 4.0/3.9; AS II: 3.7/3.5; AS III: 2.9/2.8; AS IV: 4.9/4.8; PL: 3.9/4.2; PW: 6.7/7.2; SL: 2.0/2.1; SW: 2.6/3.1.

**Distribution.** China: Beijing.

**Remarks.** The original description of this species was based on a single female specimen collected from Beijing, China, and was placed in the genus *Mictis* (Figure 14). Our study examined both male and female specimens collected from the type locality and compared them with the type specimen (Figure 14 and Figure 15). The female specimens exhibited identical morphological characters to the original description, including abdominal segments III with six black spots, confirming that our material represents the published *Mictis falloui*. However, the male morphology shows critical diagnostic differences from *Mictis*: abdomen smooth, without tubercles and hind tibia with a large spine. Conversely, male hind femora with tubercles on anterior and posterior surfaces align with the morphological characteristics of *Ochrochira*. Based on this evidence, we formally transfer this species to the genus *Ochrochira*.

## 4. Discussion

Tibial coloration cannot be used for species determination within this genus. During our examination of specimens, we also observed some transitional variations in body coloration, highlighting the importance of examining a broader range of specimens in taxonomic studies. This facilitates a better understanding of the species. Concurrently, molecular studies can offer valuable assistance. Additionally, based on our observations, coloration is not a reliable diagnostic character for species identification within the Heteroptera. We hypothesize that this may be because body color originates from pigmentary coloration, which can be influenced by more factors, such as diet and environment.

Hsiao proposed that the genus *Mictis* was primarily restricted to southern China [28], a distribution pattern largely supported by our extensive sampling across subtropical and tropical regions (Figure 2). In this study, our findings indicate that while Hsiao’s delineation generally remains valid. However, the actual distribution of this genus has reached the extreme edge of its predicted range. The genus may occasionally expand into higher latitudes through long-distance dispersal.

The tribe Mictini represents the largest group within the Coreidae and even the entire Heteroptera. However, there is considerable variation in body size among species within each genus, as well as within widely distributed species. For example, in the genus *Mictis*, based on the specimens examined in this study, the largest species, *M. arcuata*, has a body length nearly 1.5 times that of the smaller *M. tenebrosa*. In the most widely distributed species, *M. serina*, the maximum intraspecific variation in body length approaches 40%. As ectotherms, the body size of *Mictis* does not show a clear correlation with latitude or altitude. We hypothesize that, as phytophagous insects, the primary factor influencing the body size of *Mictis* is the habitat of small populations, which is also a direction for our future research.

Some Mictini species exhibit exaggerated pronotal extensions. Hsiao once used this characteristic as a key basis for dividing Mictinae (=Mictini) into two tribes [23]. The pronotum of the genus *Mictis* typically lacks significant extensions, but the new species discovered in this study currently has the most pronounced extension within the genus. Additionally, pronotal extensions in *Mictis* and most other coreid species are not associated with sexual dimorphism. The pronota of male and female individuals in this group are mostly very similar, with their dimorphism more prominently reflected in differences in the legs, particularly the hind legs. Specifically within the genus *Mictis*, this sexual dimorphism is primarily manifested in the thickened male hind femora, the structural complexity of the tibiae, and the presence of tubercles on the abdomen. Whether these modifications serve specific functions in the mating and reproduction of *Mictis* is another subject of our interest.

## Figures and Tables

**Table 1:** Uncorrected *p*-distances (%) based on the 675 bp fragment of COI among selected members of the genus *Mictis*.

		1	2	3	4
1	*M. arcuata* **sp. n.** (*n* = 2)	0.9			
2	*M. gallina* (*n* = 1)	13.2–13.3	–		
3	*M. serina* (*n* = 4)	8.2–9.0	13.3–13.8	0–1.0	
4	*M. tenebrosa* (*n* = 2)	11.7–12.1	10.2	13.3–13.6	–

## Data Availability

The original contributions presented in this study are included in the article. Further inquiries can be directed to the corresponding author.
